# Concurrent Associations Between the OMNI Rating of Perceived Exertion and Measures of Monitoring Training Load in Non-Elite Adolescent Team Sport Athletes

**DOI:** 10.3390/sports14080332

**Published:** 2026-08-03

**Authors:** David Sainsbury, Leanda McKenna, Jenny Downs, Kevin Netto

**Affiliations:** 1 School of Allied Health, Curtin University, Bentley, Perth 6102, Australia; l.mckenna@curtin.edu.au (L.M.); kevin.netto@curtin.edu.au (K.N.); 2 The Kids Research Institute Australia, Perth Children’s Hospital, Nedlands, Perth 6009, Australia; Jenny.Downs@thekids.org.au

**Keywords:** adolescent, team sport, rating of perceived exertion, training load

## Abstract

This study aimed to investigate associations between training load (TL) calculated using the OMNI rating of perceived exertion scale (OMNI-RPE) and heart rate (HR) and velocity-derived measures of TL in non-elite adolescent team sport athletes. Fifty-four athletes participated in the study. The study was separated into two parts. In the Part A, twenty athletes completed both an easy and hard version of a simulated team game circuit outdoors on natural turf to replicate performance parameters often used in training. In part B, thirty-four different athletes completed a competitive team game played outdoors on synthetic/natural turf hockey or soccer pitches. For both Parts A and B, session OMNI TL (sOMNI-TL) was derived as a product of the session OMNI-RPE and session duration, and was correlated to Edward’s Training Impulse (TRIMP) and time spent in 0–25%, 25–50%, 50–70% and >70% peak velocity. Low-to-moderate correlations were observed between sOMNI-TL and Edwards TRIMP (r = 0.18–0.20) and velocity measures (r = 0.05–0.42) for Part A. Larger correlations were observed between sOMNI-TL and Edwards TRIMP (r = 0.53, *p* < 0.05) and velocity measures (r = 0.36–0.51, *p* < 0.05) for part B. The hypothesis that sOMNI-TL would show significant, moderate associations with heart rate and velocity-derived training load in both simulated and game conditions was only partially supported, and the results of this study do not support its use as a stand-alone measure in a practical setting.

## 1. Introduction

High training load is a potential risk factor for injury in the non-elite adolescent team sport [1]. Typically, non-elite adolescent sports clubs do not have access to sophisticated methods to monitor their players’ training loads; so, accessible, affordable and developmentally appropriate methods to monitor training load are essential [2]. Session rating of perceived exertion training load (sRPE-TL) is a readily accessible method to monitor training load. Further rigorous research investigating its utility in non-elite adolescent athletes with the use of paediatric specific RPE scales, such as the OMNI rating of perceived exertion (OMNI-RPE) scale [3], is needed, however [2].

Session rating of perceived exertion training load is derived by calculating the product of a session’s duration and the rating of perceived exertion (RPE) score typically from a Borg Category Scale with Ratio Properties (CR-10) [4] or a Borg centiMax (CR-100) scale [5] for that exercise session [6]. This provides a sRPE-TL measure, which is shown to be strongly correlated with heart rate indices and global navigation satellite systems (GNSS)-derived measures of training load in adult team sport settings [7,8]. Determining the relationships of sRPE-TL against other internal measures of load is important as these measures provide a representation of the internal physiological responses occurring. Furthermore, determining relationships between sRPE-TL and external measures of training load is important as most team sport conditioning programmes are prescribed in terms of external load, such as running intensity and distance covered. The sRPE-TL method to monitor training load has been used effectively in adult team sports to help inform potential injury risk whereby appropriate safe doses of training loads and progression have been identified [9].

Rating of perceived exertion is moderated by cognitive factors, memory and previous experience; therefore, simplified pictorial scales of perceived exertion have been developed for use in child and adolescent populations which are matched to verbal anchors of perceived effort [3,10,11]. Pictorial scales may be developmentally more appropriate to use with younger athletes, compared to verbally anchored Borg scales, as they may find it difficult to assign a number or a phrase to perceived exercise exertion [12].

**Figure 1:**
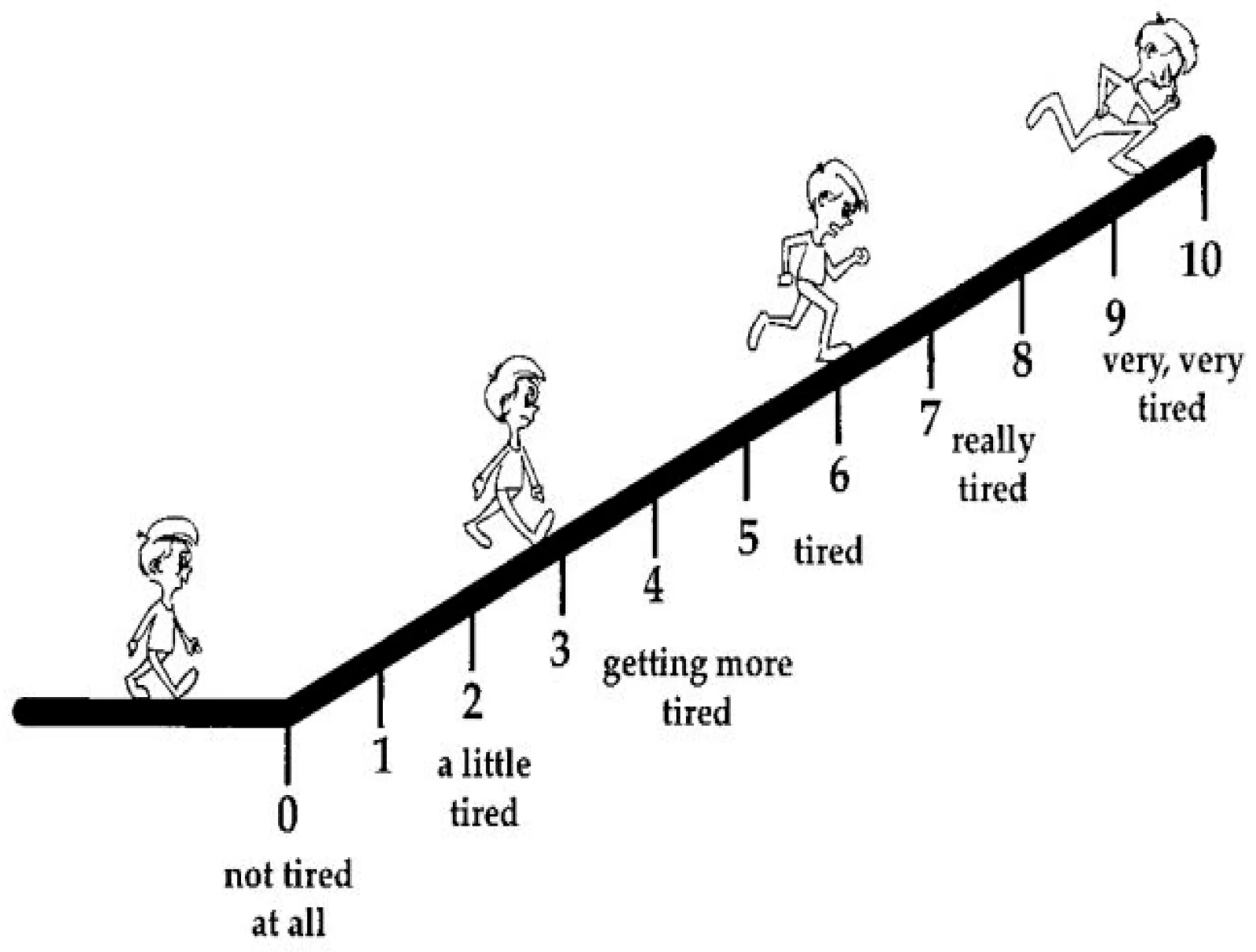
Children’s OMNI scale of perceived exertion for walking/running. From Robertson RJ, Goss FL, Boer NF, Peoples JA, Foreman AJ, Dabfromeh IM et al. Children’s OMNI scale of perceived exertion: mixed gender and race validation. Med sci sports exerc. 2000;32(2):452–8 [3].

One such paediatric specific RPE scale is the OMNI-RPE scale [3]. (Figure 1). This is a pictorial scale categorised by scores and verbal links ranging from 0 “not tired at all” to 10 “very, very tired”. The OMNI-RPE scale was designed for use with children and adolescents of mixed ethnicity and is developmentally appropriate [3,11]. The OMNI-RPE scale has been shown to be a valid tool to determine exercise intensity in adolescents, when used in controlled steady state environments and compared against heart rate indices and oxygen uptake [3,11]; however, there appears to be limited research evaluating is its utility in the field. Furthermore, The OMNI-RPE scale has also demonstrated concurrent validity in children as young as eight years of age in light-to-moderate intensity lifestyle activities and moderate to vigorous running, aerobic and continuous basketball drills when compared against heart rate and oxygen consumption measures [13,14]. Therefore, the OMNI-RPE scale was conceptually appropriate for use with the population used in this study. It is important to acknowledge, however, that a familiarisation period over a number of weeks in various game and training scenarios is required for improved reliability with younger athletes [15].

Similar to Borg scales, the OMNI-RPE scale can also be used to derive a session OMNI training load (sOMNI-TL) by calculating the product of a session’s duration and the OMNI-RPE for that exercise session.

A scoping review on the use of RPE scales in children and adolescents concluded that the utility of RPE scales to monitor training load in this population remains unclear due to the small number of studies using paediatric-specific scales such as the OMNI-RPE scale [15].

Although a recent meta-analysis reported high correlations between session rating of perceived exertion training load (sRPE-TL) and heart rate-derived measures of training load in adolescents [16], the strength of this conclusion is limited by the characteristics of the included studies. Only one of the 16 included studies used a paediatric-specific perceived exertion scale (OMNI-RPE). This is an important limitation because adult-derived Borg scales may not adequately reflect how younger athletes interpret and report effort. Furthermore, only two of the 16 studies in the meta-analysis included non-elite team sport participants [16]. Findings drawn largely from elite cohorts may not be directly transferable to non-elite adolescents, who typically have less specialised training histories, lower exposure to structured competition, and potentially less developed perceptual awareness of exercise intensity [17,18].

The limited evidence in non-elite adolescent team sport populations is inconsistent. Of the two available studies, authors found both low [19] and high [20] correlations between sRPE-TL and the Edwards TRIMP in younger [19] and older [20] adolescents during training sessions who play non-elite team sports. These differences could relate to the differences in athlete age, the type of RPE scale type used, sports played, or training session structure.

Furthermore, there appears to be an absence of research examining associations between sRPE-TL and velocity-derived measures of training load, and there appears to be limited evidence evaluating Borg or paediatric-specific session RPE scales during competition exposure in the non-elite adolescent team sport setting. Addressing these gaps is important because competition exposure may impose distinct physical and cognitive demands and is recognised as a potential injury risk factor in adolescent athletes [1].

Recognising the importance of accurately monitoring training load in competition, a step-wise process would be to first investigate associations between sRPE-TL and heart rate and velocity-derived measures in a simulated team game setting, and then in an actual game setting. Simulated team game scenarios incorporating repeated sprint efforts, active recovery, and rest over a period of time are often used to replicate performance parameters [21].

The aim of this study was to investigate associations between the OMNI rating of perceived exertion and heart rate- and velocity-derived measures of monitoring training load in non-elite adolescent team sport athletes in field-based settings. We hypothesised that sOMNI-TL would show significant, moderate associations with heart rate and velocity-derived training load in both simulated and game conditions. An easily applied method to monitor training load in this population may inform coaches and players of potential injury risk where access to more sophisticated measures may be limited.

## 2. Materials and Methods

This study was conducted accordance with the Declaration of Helsinki and ethical approval was obtained from Curtin University Human Research Ethics Committee (HRE2020-0739) on 10 December 2020. Informed written consent was obtained from each participant and their legal guardian prior to the commencement of their participation in the study. The information collected in this research was coded to remove identifying information on any data. Data from this study is stored in a secure repository until the subjects have reached 25 years of age when it will be destroyed.

### 2.1. Participants

A total of fifty-four athletes participated in the study. Athletes were included if they were aged 10–18 years old and participated in an invasive team sport at a non-elite level. Invasive team sport was defined as an activity or game in which a team compete physically against another team in a common field to gain territorial dominance and score against that team [22]. Elite level was defined as competing at state, regional or national level, or playing at the highest level of competition against intercity, interstate or international teams or being selected for a youth academy or talent development program linked with a first- or second-tier professional club [1]. Athletes were excluded if they had a self-reported chronic health condition that prevented them from performing physical activity at a vigorous intensity associated with the team sport. Athletes were recruited through junior Australian rules football, soccer, and field hockey community clubs based within Perth, Western Australia. Prior to testing, each athlete’s date of birth, sex, sports played and years playing each sport were recorded by researchers using a questionnaire. Each athlete’s height (cm) and weight (kg) were also measured and recorded by the researchers using a calibrated stadiometer and weighing scales. The athletes were cleared to perform vigorous exercise testing with the Youth Physical Activity Readiness Questionnaire [23] along with discussion with the athletes and their caregivers.

For Part A—Simulated team game circuit—twenty athletes were recruited with a mean age of 14.2 ± 1.7 (range 11.8–17.8) years, height of 1.68 ± 0.09 (range 1.49–1.87) m, and mass of 56.3 ± 11.6 (range 41.0–82.0) kg. Twelve athletes were male. The athletes either played only Australian Rules football (7 males, 35%), Australian Rules football and basketball (3 males, 15%), only netball (2 females, 10%), only hockey (2 females, 10%), Australian Rules football and netball (1 female, 5%), netball and tee-ball (1 female, 5%), hockey and basketball (1 male, 5%), hockey and softball (1 female, 5%), soccer and netball (1 female, 5%) and basketball and cricket (1 male, 5%) and had been playing team sports for 5.95 ± 3.58 (range 1–14) regular seasons, and currently trained ≥2 times per week.

For Part B—Team game—thirty-four athletes (different to those in Part A) were recruited from a male soccer team, a male hockey team and two female hockey teams, age: 15.2 ± 1.5 (range 11.8–17.9) years, height: 1.73 ± 0.07 (range 1.56–1.88) m, mass: 59.7 ± 10.6 (range 36.0–81.0) kg. Eleven male athletes were analysed over the duration of their soccer game, 7 male athletes were analysed over the duration of their hockey game, and 16 female athletes were analysed over the duration of two separate hockey games. Goal keepers were excluded as their training load differs from outfield players [24]. All athletes trained ≥2 times per week.

Separate groups of athletes were used for Part A and Part B of this study due to logistical constraints and to reduce participant and administrator burden in the non-elite community club setting.

### 2.2. Training Load Measures

OMNI-TL expressed in arbitrary units (au), was calculated as a product of the OMNI-RPE for a session and the session duration in minutes according to the following formula:

sOMNI-TL(au) = OMNI-RPE for session × session duration (min)

All athletes were taught how to use the OMNI-RPE scale using a paper copy of the scale and according to standardised instructions [3] prior to testing. Athletes OMNI-RPE was measured 30 min after the completion of the session to remove transient affective bias ensuring the most recent exercise intensity would not dominate the athletes rating for the entire session [6].

Other internal measures of training load included the Edwards TRIMP, expressed in au. The Edwards TRIMP was used as it has been used in previous concurrent validity studies [16] and is a valid and reliable measure of internal training load [25,26]. It was calculated by multiplying exercise duration (in minutes) in each of the five heart rate zones by a numerical factor relative to that zone (50–60% peak HR = 1, 60–70% peak HR = 2, 70–80% peak HR = 3, 80–90% peak HR = 4 and 90–100% peak HR = 5), then summating the results according to the following formula:

Edwards TRIMP (au) = [∑ time (min) spent in zone × numerical factor of zone]

External measures of training load included time spent in low-speed activities (0–25% of relative peak velocity), time spent in moderate-speed running (25–50% of relative peak velocity) time spent in high-speed running (50–70% of relative peak velocity) and time spent in very-high-speed running (>70% of relative peak velocity) expressed in minutes (min). These relative velocity zones were based on a study of junior rugby league players where relative rather than absolute velocity zones were found to minimise over- or underestimation of time spent in different zones when athletes of differing ages and abilities were studied [27]. Furthermore, the research involving junior rugby league players possesses ecological validity, making its findings applicable to the invasive team sports of hockey and soccer investigated in this study.

### 2.3. Procedures

This study had two parts, Part A and Part B.

In Part A, participants completed a simulated team game circuit, where athletes performed both easy and hard versions of a circuit designed to replicate the physical demands of team sport in a controlled environment [21]. The circuit involves repeated maximal sprint efforts, agility and jumping tasks and longer periods of lower speed running and active recovery. In Part B, different athletes to those in Part A performed their usual team game in a competition environment along with a coach-prescribed warm up (running- and skill-related drills for approximately 30 min prior to the game) and cool down at the end of a player’s game (slow walking/jogging for approximately 30 min).

**Figure 2:**
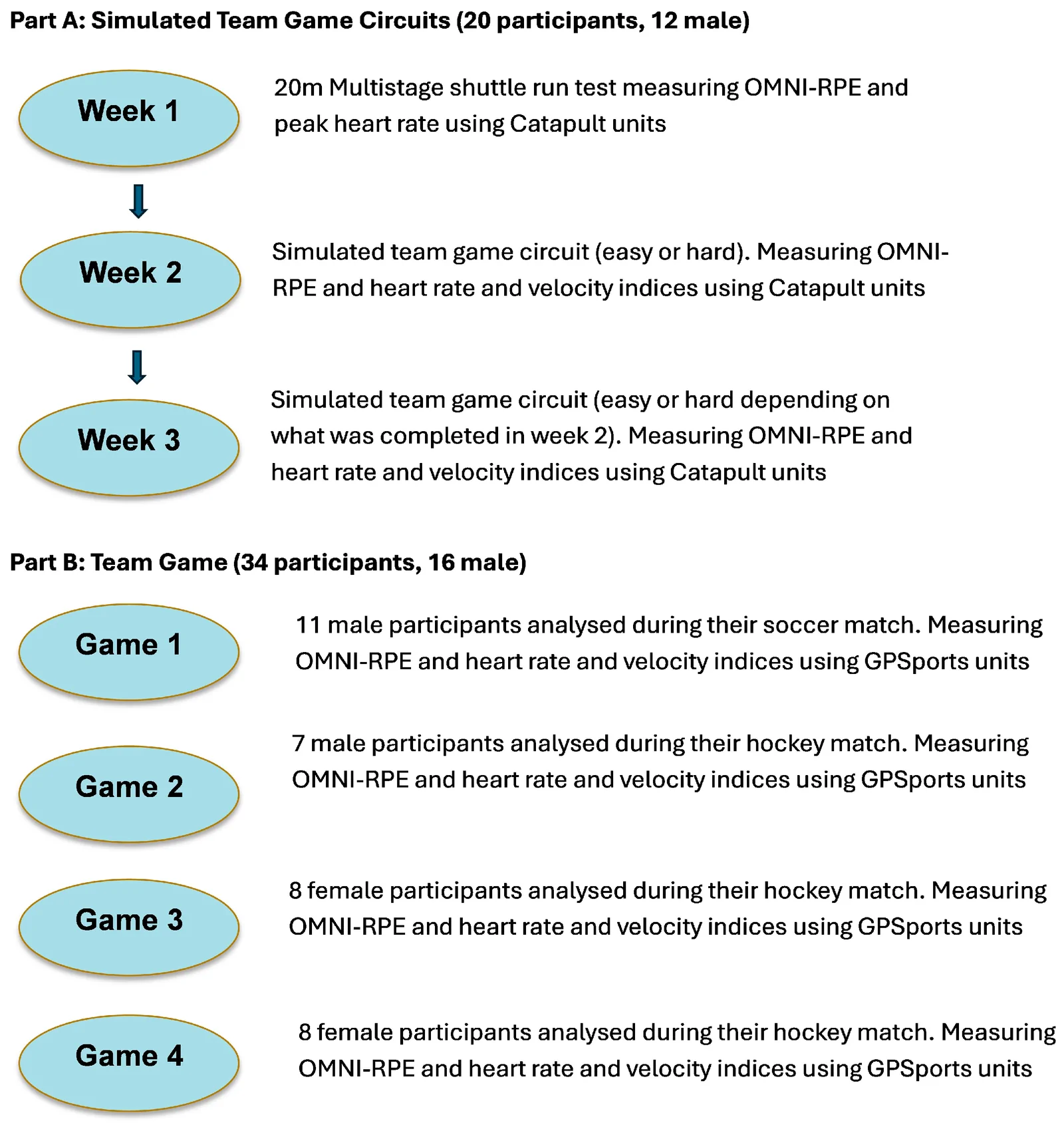
Flow diagram summarising timeline and measurement instruments used for both Part A and Part B.

Both Part A and Part B in this study separately used a within-subject design where the athletes were tested across different independent exercise conditions, i.e., easy version of the circuit, hard version of the circuit for Part A and a naturalistic team game setting for Part B. A flow chart summarising the timeline and testing procedures for both Part A and Part B is presented in Figure 2.

### 2.4. Part A—Simulated Team Game Circuit

**Figure 3:**
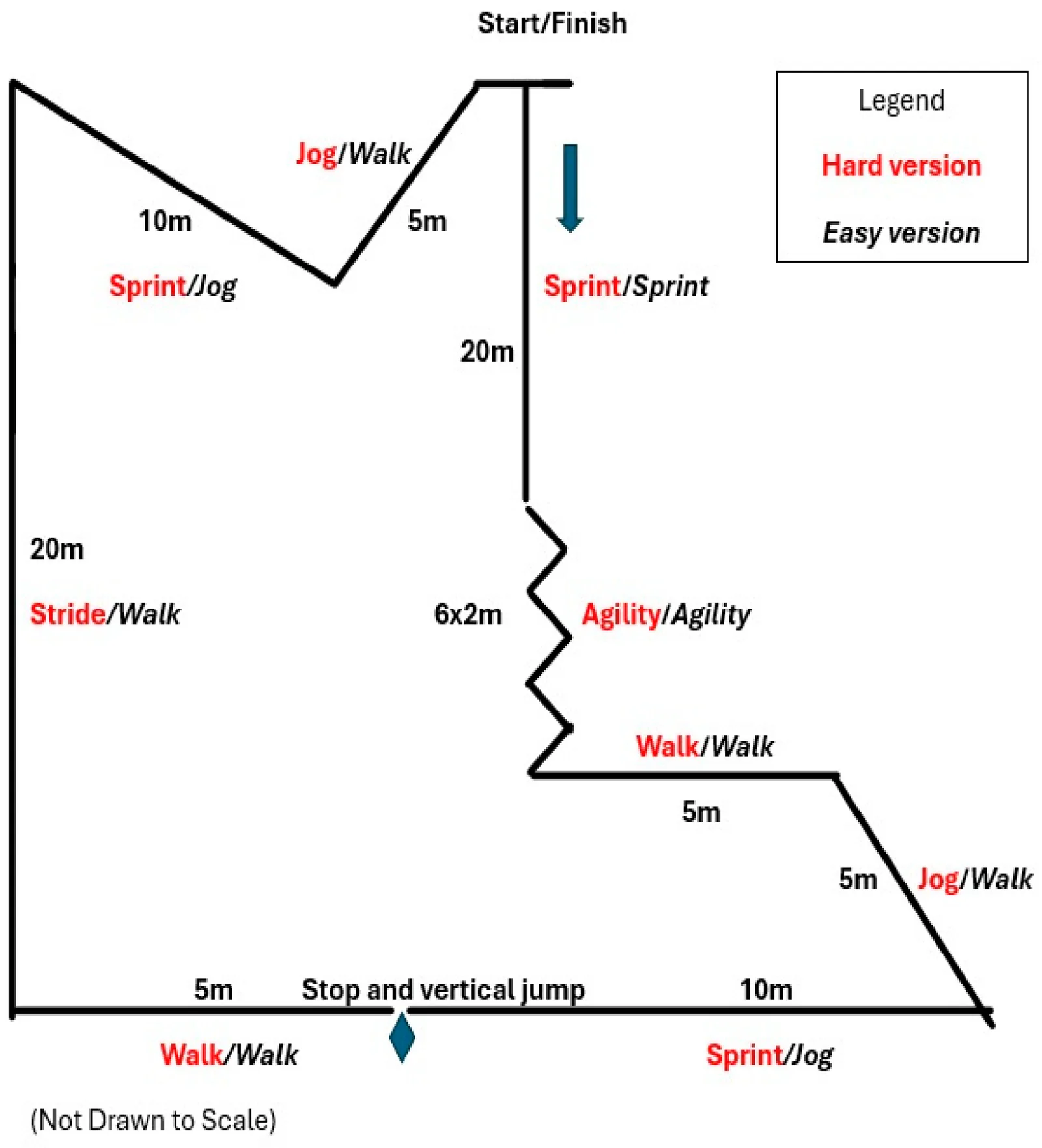
Hard and easy versions of the simulated team game circuit. Adapted from Singh TKR, Guelfi KJ, Landers G, Dawson B, Bishop D. Reliability of a contact and non-contact simulated team game circuit. Journal of Sports Science & Medicine. 2010;9(4):638–42 [21].

The original simulated team game circuit was designed for conditioned adult team sport athletes and involved four sets of 15 min running of the circuit with a five-minute break between circuits. For this study, this protocol was piloted on three team sport players; two male soccer players aged 16.3 years and 15.5 years and one female netball player aged 13.5 years. These players were unable to complete the entire circuit, so it was modified to two sets of 5 min intermittent running of the circuit with a 5 min break between circuits. It was further modified to a hard and easy version by differentiating speeds at different sections of the circuit and evaluating the pilot participants RPE, heart rate, and velocity responses. (Figure 3). The easy and hard versions in this study were used as a surrogate to exert practice match training loads on athletes to simulate easy and hard game scenarios where a mismatch of teams can occur in community sport such that one team gets an easy game and wins by a large margin.

Athletes in Part A were equipped with a GNSS-enabled Catapult Optimeye S5 units (Catapult Sports, Melbourne, Australia) and a compatible heart rate unit (T31, Polar Electric, Kempele, Finland) sampling at 10 Hz and capturing information on velocity and heart rate. These units have demonstrated validity when compared to criterion measures of maximum sprint speed (mean difference −0.84% between criterion measure) and reliability (intraclass correlation coefficient 0.98, typical error of measurement 0.02) in returning GNSS measures in male and female adult rugby and ice hockey players [28]. The Optimeye S5 units were worn in a padded pouch in a custom-made vest (Catapult Sports, Melbourne, Australia) and the Polar heart rate units were worn with an adjustable custom-made strap placed around the athletes’ lower chest. The velocity and heart rate measures were extracted using Catapult software version 5.1.7 to determine athletes’ Edwards TRIMP and time spent in 0–25%, 25–50%, 50–70% and >70% peak velocity.

Heart rate zones were determined based on the athletes’ peak heart rate measured during a 20 m multistage shuttle run test [29]. The multistage shuttle run test is reliable and will achieve maximum effort to volitional fatigue when used in child and adolescent populations in naturalistic environments [29,30].

Velocity zones were determined based on athletes’ fastest maximal sprint measured in the initial 20 m sprint zone of the simulated team game circuit after coming in from either a 5 m walking or rolling jogging start, as seen in Figure 3. This distance was used as sprint speed testing for field-based team sport athletes should specifically concentrate on acceleration over the first 20 m [31] which replicates the importance of developing speed over shorter distances required in a game scenario [32,33]. For athletes under 12 years of age, maximum sprint speed can be achieved in under 20 m, and athletes between the ages of 14 and 16 years require 20–30 m; however, this is from a standing start [34]. Velocity was measured using GNSS technology. GNSS units (with sampling rates ≥ 10 hz) show strong agreement with criterion measures of peak velocity (radar or laser) with small standardised mean differences between the two measures [35].

Testing was conducted over three sessions, each one week apart. Session one was the 20 m multistage shuttle run to determine the athletes peak heart rate at volitional fatigue and was conducted in an indoor, climate-controlled wooden floor multisport court. Directly after the completion of the 20 m multistage shuttle run, the athletes’ OMNI-RPE were recorded directly by the researchers. Sessions two and three were conducted on outdoor natural turf sports pitches. For session two, athletes were allocated to complete either a hard or an easy version of the simulated team game circuit by the investigator. For the session three, athletes completed the version of the circuit which they had not completed in session two (easy or hard). For both the easy and hard sessions athletes completed a standardised 10 min warm up (Appendix A), 5 min running the circuit, 5 min rest, 5 min running the circuit again and finishing with a standardised 10 min cool down (Appendix A). For sessions two and three, each athlete’s session OMNI-RPE was recorded via phone short message service (SMS) 30 min after the completion of the session where athletes had their own paper copy of the OMNI-RPE scale.

### 2.5. Part B-Team Game

Athletes in Part B were equipped with a GNSS-enabled GPSports XPI Pro X units (GPsports, Canberra, Australia) and a compatible heart rate unit (T34, Polar Electric, Kempele, Finland) sampling at 15 Hz capturing information on velocity and HR, which have demonstrated validity when compared to criterion measures of maximum sprint speed (absolute difference between criterion measure 0.09) and reliability (coefficient of variation 0.11%, typical error of measurement 0.11) in returning GNSS measures in male adult American football players [36]. We reverted to the GPSports units in Part B due to the availability of functional Catapult units needed for team monitoring. Sampling rates for both devises used are ≥10 hz, which demonstrates strong agreement with criterion measures of velocity, and since this study adopted a within-subject design, this minimises the potential systemic error that may arise from using different GNSS units. The GPSports units and Polar heart rate units were worn as described in Part A of the study. Velocity and HR measures were then extracted using GPSports software (version P10) to determine athletes’ Edwards TRIMP and time spent in 0–25%, 25–50%, 50–70% and >70% peak velocity. Heart rate zones were determined based on athletes’ peak heart rate measured during the team game and velocity zones were determined based on athletes’ fastest maximal sprint (metres per second) measured during the team game using GNSS technology.

Testing was conducted over the duration of four independent team games (one soccer game and 3 hockey games), which consisted of a coach-directed warm up, periods of play (20 min half game periods for soccer and 15 min quarter game periods for hockey), breaks between periods of play, and a coach-directed cool down. The soccer games were conducted on a standard size natural turf pitch (105 m × 68 m) with 11 players on field, and the hockey games were conducted on a standard artificial turf pitch (91 m × 55 m) with 11 players on field. Each athlete’s OMNI-RPE was recorded 30 min after the completion of the game directly by the researchers.

### 2.6. Statistical Analysis

All statistical analysis were conducted using the statistical software IBM SPSS Statistics for Windows (Version 29, IBM corporation, Armonk, NY, USA) with a *p* < 0.05 alpha level of significance. The assumption of normality was assessed using the Shapiro–Wilk test. Homoscedasticity was assessed using a modified Breusch–Pagan test, which is an appropriate test in the presence of outliers [37]. Linearity was assessed using the ANOVA test for linearity of paired associations. For all normally distributed data, descriptive statistics were expressed as mean and standard deviation (SD) values. To evaluate whether there were statistically significant differences in participants’ variables measured between the hard and easy simulated team game circuits, paired *t* tests were used. To assess data dispersion, coefficients of variation were used. Relationships between variables were independently determined using two-tailed bivariate Pearson product–moment correlation (expressed as r), along with a 95% confidence interval. For data that does not meet Pearson product–moment assumptions of normality, linearity and homoscedasticity, a Spearman rank correlation (expressed as ρ), along with a 95% confidence interval was used. A sample of *n* = 20 has 80% power to show a minimum correlation coefficient of 0.52 in a bivariate correlation, (alpha = 0.05, one-tailed). A correlation coefficient greater than r = 0.5 was chosen for the a priori power analysis because it is within the range of those reported in initial walking/running association studies of the OMNI scale with heart rate, oxygen consumption and ventilation measures [11].

Missing data was managed by pairwise deletion. The size of the correlation was interpreted as: r < 0.1 trivial, r = 0.1–0.3 low, r = 0.3–0.5 moderate, r = 0.5–0.7 large, r = 0.7–0.9 very large, r > 0.9 nearly perfect and r = 1 perfect [38].

## 3. Results

### 3.1. Part A—Simulated Team Game Circuit

The athletes OMNI-RPE scores were higher in the 20 m multistage shuttle run test and the hard simulated team game circuit, as were the athletes peak heart rates (Table 1). Low coefficients of variation were also observed in peak heart rate measures during the easy and hard simulated circuits and the team game.

Compared to the easy version of the circuit, in the hard version participants had a higher OMNI-RPE (*p* < 0.001), sOMNI-TL (*p* = 0.021), peak heart rate (*p* = 0.042), Edwards TRIMP (*p* = 0.021) and time spent in >50–≤70% peak velocity (*p* = <0.001). In the hard version participants spent less time in 0–25% peak velocity (*p* < 0.001) and >70–100% peak velocity (*p* = 0.012) and there was no difference in the time that participants spent in >25–≤50% peak velocity (*p* = 0.069).

For the easy and hard versions of the circuit, trivial-to-moderate correlations were observed between sOMNI-TL and heart rate- and velocity-derived measures of training load, (r = 0.05 to r = 0.42) with the highest correlations occurring between sOMNI-TL and time spent in moderate-speed running (25–50% of peak velocity) and high-speed running (50–70% of peak velocity). These correlations did not reach significance thresholds of *p* < 0.05 (Table 2).

For the easy simulated team game circuit, one athlete dropped out and GNSS data is missing for one athlete in both the easy and hard simulated team game circuits.

### 3.2. Part B: Team Games

The athletes peak heart rates were close to expected maximum for this age group (Table 1) [39]. Moderate-to-borderline-large significant correlations (*p* < 0.05) were found between sOMNI-TL and heart rate and velocity-derived measures of training load (r = 0.36 to r = 0.53) (Table 2). For the team game, heart rate data is missing for one athlete and GNSS data is missing for one athlete.

For the team game, the majority of time was spent in low- (0–25% peak velocity) and moderate- (25–50% peak velocity) speed running, with the least time spent in very-high-speed running (>70% peak velocity) (Table 1), and correlations with OMNI-TL were the lowest for the time spent in very-high-speed running (>70% peak velocity) (r = 0.36) (Table 2).

## 4. Discussion

Session OMNI training load showed trivial-to-moderate correlations with HR- and velocity-derived measures of training load in simulated drills and moderate-to-borderline large correlations in match play. The small sample size, particularly in Part A, and wide confidence intervals reflect high variability in the results and uncertainty in any significant correlations reported. Based on the findings of this study, it would be imprudent to recommend session OMNI training load as a standalone measure of training load in community team sport settings.

For Part A, the simulated team game circuit, low-to-moderate correlations were observed between sOMNI-TL and the heart rate- and velocity-derived measures of training load. This compares similarly with previous research in both adolescent and adult team sport settings where lower correlations between heart rate-derived internal measures of training load and sRPE-TL have been observed in sessions which are more intervalised, anaerobic and skill-based in nature. A study with non-elite adolescent soccer players found low correlations between Edwards TRIMP and training load derived from the OMNI-RPE during intermittent technical training sessions [20]. Similarly, a study with adult female soccer players found that Bannister TRIMP and Edwards TRIMP correlated higher with sRPE-TL in less intermittent, more aerobic-based training sessions [40]. Another study with adult female soccer players also found correlations between sRPE-TL and internal heart rate-derived and external GNSS-derived measures of training load were higher during match play, compared to training sessions [7].

One reason for the lower correlations in this study between session RPE training load and the heart rate-derived internal measures of training in the simulated circuits might be because heart rate-derived methods focus on aerobic effort, not accurately capturing the high-intensity bursts and explosive movements which can also contribute to perceptions of exertion. The simulated circuits analysed in this study consisted of repeated maximal sprint efforts with longer periods of lower speed running, active recovery between circuits and a relatively long warm up and cool down in relations to the overall circuit time. Thus, the time lags observed between heart rate values from explosive movements and sprints are diluted by prolonged lower intensity activities and rest periods [8].

Lower correlations were also observed between sOMNI-TL and time spent in very-high-speed running (>70% peak velocity). Although there are no adolescent team sport studies on which to make comparisons, this finding is similar to what has been observed in elite youth and adult studies. Low correlations between sRPE and the fastest velocity zones were observed in training (r = 0.15) and games (r = 0.15) with National Collegiate Athletic Association (NCAA) female soccer players [7], and moderate correlations were also observed between sRPE and high-speed running distance per minute (r = 0.39) in elite youth soccer players (age 17.81 ± 0.96) during training compared to other velocity zones [41]. The lower correlations observed in the current study at higher velocity zones may also, in part, be explained by a dilution effect, as a relatively small amount of time was spent at very high speeds, compared to the time spent at low-to-moderate speeds and with prolonged rest periods.

For Part B, the team game, the correlation with Edward’s TRIMP in this study (r = 0.53) is lower than what has been found in adolescent team sport studies involving elite mid-to-late adolescent soccer players, which have used either the CR-10 scale or the CR-100 scales to derive training load (r = 0.64 to r = 0.84) [8,24,42,43]. This, in part, may theoretically be due to scale categorisation and differences in alignment with metabolic cues between the Borg and OMNI-RPE scales. In the CR-10 scale, scores of five at the middle of the scale are appointed to a strong perception of exertion which may correspond to anaerobic threshold [44]. By contrast, the middle of the OMNI-RPE scale is appointed for more moderate level of tiredness and “tired” is six.

The lower correlation between sOMNI-TL and Edwards TRIMP in this study compared to previous studies may also be attributed to the use of the OMNI-RPE anchor of “tiredness” compared to Borg scales’ anchor of “exertion” which may better correlate with physiological measures of exercise intensity [43]. The OMNI-RPE anchor of “tiredness” could be perceived as an unpleasant sensation of fatigue or weariness, reflecting an athlete’s overall subjective interpretation of exercise symptoms, integrating multiple factors such as respiratory effort, sweating, and psychosocial factors like mood states, cognitive load and performance-related stressors [15,45,46]. The OMNI-RPE scale, when used in a competition setting, may be potentially more sensitive to the factors that contribute to “tiredness”, such as cognitive, tactical and performance-related stressors. Given that constructs of tiredness and exertion were not directly measured in this study, this conceptual distinction between tiredness and exertion remains speculative.

Interpretation of exertion is also influenced by athletic experience and to accurately rate perceived exertion, an athlete needs cognitive maturation to recall what it is like to experience volitional exhaustion, and past experiences of exercise at different intensities [47]. Although there are no adolescent team sport studies which explore the associations between perceived exertion and athletic experience, a study with adolescent swimmers found that the more experienced athletes were better at perceiving effort compared to their less experienced counterparts, presumably due to greater exposure to variable intensities of exercise over their years of training [19]. The lower correlation between sOMNI-TL and Edwards TRIMP observed in this study may be partly explained by participant characteristics. Unlike previous adolescent team sport studies which involved elite athletes with potentially greater training and monitoring experience, the present study included non-elite athletes who may have had less experience interpreting and using the scale. In addition, participants did not complete a familiarisation period over a number of weeks before using the measure, which may have further influenced the strength of the relationship between the two training load metrics.

### 4.1. Practical Implications

Although the moderate significant correlations observed between sOMNI-TL and Edwards TRIMP and velocity-derived measures of training load suggest sOMNI-TL could potentially have utility in monitoring training load in non-elite adolescent team sport settings during competition, the uncertainty in the results of this study, based on the small sample size and wide confidence intervals reflecting high variability in the results, doesn’t support its use as a stand-alone measure in a practical setting. This uncertainty is further compounded by the fact that inexperienced younger athletes’ perception of exertion may be unreliable. For these reasons sOMNI-TL should not be used as stand-alone measure in the non-elite community setting. It is recommended that if RPE measures of training load are used in adolescent sports, they should be complimented by other accessible measures of training load [48]. In the non-elite team sport setting, where access to sophisticated measures of training load monitoring may be limited, young athletes should be encouraged to keep training diaries to monitor their training loads, health and well-being [48]. Regular monitoring of these diaries by young athletes, caregivers and coaches would help provide an individualised understanding of how training loads might influence attendance, performance, and injury risk.

### 4.2. Future Research

Future research could investigate longitudinal studies of association between the OMNI-RPE scale and other methods of measuring training load over the course of a season, particularly when seasonal variations such as conditioning and competition exist. Collecting data through multiple training sessions or game exposures would also better account for variability and scale familiarisation, particularly with athletes who may be less experienced in interpreting and using RPE scales. Furthermore, studies into intra-individual reliability would be beneficial, particularly in the non-elite population where perception of effort may be less precise compared to elite athletes. To further investigate differences between the OMNI-RPE and Borg scales, comparisons between training load obtained using both scales within the same cohort of athletes could also be made.

### 4.3. Strengths and Limitations

The broad and diverse adolescent team sport population in this study adds strength to the generalisability of the results across different invasive field sports.

A smaller sample size may be a limitation, particularly in the Part A of this study. This may be more pertinent since pairwise deletion was used to account for missing data, resulting in no data for two athletes in the easy simulated team game circuit, no data for one athlete in the hard simulated team game circuit, and no data for two athletes in the game analysis. Furthermore, smaller but potentially meaningful associations may not have been detectable due to statistical power. The small sample size potentially resulted in wider confidence intervals, particularly when parametric tests of association were used, potentially reflecting uncertainty in significant correlations reported. For these reasons, the current study should be regarded as an exploratory study.

The use of different GNSS units for Part A and Part B is also recognised as a limitation of the study. Although sampling rates for both devises used are ≥10 Hz, which demonstrates strong agreement with criterion measures of velocity, the difference in sampling rates between GNSS units limit direct comparisons between Part A and Part B. Furthermore, as different participant groups were used in Part A and Part B, any comparisons between groups need to be made with caution.

Although this study used relative heart rate zones based on the athletes measured peak heart rate, only Part A measured this with a standardised incremental test to volitional fatigue. Being a pragmatic study with non-elite team sport athletes, we measured peak heart rate in the game setting. Peak heart rates are found to be higher in competition than in laboratory testing possibly due to an increased state of arousal [49], which needs to be considered with this study, however peak heart rates of the athletes were close to a theoretical maximum [38] with a low coefficient of variation (0.04). Also, being a pragmatic study, peak velocity was measured in a field setting during maximal sprint efforts in the simulated circuits (Part A) or during the team game (Part B). Although adolescent team sport athletes peak match velocities are close to their measured maximal sprint velocities, peak match velocity can be affected by the position they play [50]. This may result in an under-or overestimation of the time in certain velocity zones for some of the athletes in Part B of this study.

## 5. Conclusions

The OMNI-RPE-derived session training load measure shows moderate associations relative to heart rate- and velocity-based indices during natural adolescent team games but weak associations during brief, intermittent simulations. The uncertainty in the results of this study do not support its use in a practical setting by coaches and athletes as a stand-alone measure of training load in the non-elite adolescent team sport population, particularly considering that inexperienced younger athletes’ perception of exertion may be unreliable.

## Figures and Tables

**Table 1:** Descriptive statistics for session OMNI-RPE, sOMNI training load, Edward’s TRIMP, peak heart rate, peak velocity and time spent in 0–25%, >25–≤50%, >50–≤70% and >70–100% peak velocity for the 20 m multistage shuttle run test, easy and hard simulated team game sessions and the team game session.

	OMNI-RPE Mean ± SD CV (Min–Max)	sOMNI-TL (au) Mean ± SD CV (Min–Max)	Edward’s TRIMP (au) Mean ± SD CV (Min–Max)	HR Max (bpm) Mean ± SD CV (Min–Max)	PV (m/s) Mean ± SD CV (Min–Max)	Time Spent in 0–25% PV (min) Mean ± SD CV (Min–Max)	Time Spent in >25–≤50% PV (min) Mean ± SD CV (Min–Max)	Time Spent in >50–≤70% PV (min) Mean ± SD CV (Min–Max)	Time Spent in >70–100% PV (min) Mean ± SD CV (Min–Max)
20 m multistage shuttle run test	8.5 ± 1.50 0.18 (6–10)	-	-	206.4 ± 7.82 0.04 (193–222)	-	-	-	-	-
Easy simulated team game ^1^	5 ± 1.47 0.29 (2–8)	174.9 ± 52.3 0.30 (65.9–277.9)	82.3 ± 23.8 0.29 (19.6–121.0)	191.05 ± 13.57 0.07 (161–209)	6.48 ± 0.49 0.08 (5.43–7.19)	27.06 ± 1.79 0.07 (24.04–29.67)	6.06 ± 1.30 0.21 (4.26–7.86)	0.91 ± 0.54 0.59 (0.32–0.76)	0.6 ± 0.14 0.23 (0.4–0.9)
Hard simulated team game ^2^	7 ± 1.02 0.15 (5–9)	237.3 ± 37.6 0.16 (174.8–314.7)	95.1 ± 17.5 0.18 (77.4–123.6)	197.73 ± 12.68 0.06 (168–217)	6.23 ± 0.51 0.08 (5.40–7.31)	25.69 ± 1.44 0.06 (22.96–27.42)	6.64 ± 1.19 0.18 (4.27–8.33)	2.36 ± 0.85 0.36 (0.92–4.50)	0.5 ± 0.3 0.60 (0.2–0.8)
Team game ^3^	5.50 ± 1.62 0.29 (2–8)	467.42 ± 148.05 0.31 (188.00–714.26)	271.74 ± 75.75 0.28 (104.57–393.99)	201.49 ± 7.55 0.04 (189–213)	6.56 ± 1.08 0.19 (4.80–8.90)	62.81 ± 9.04 0.14 (45.54–80.55)	12.98 ± 5.04 0.39 (4.99–23.28)	4.07 ± 2.21 0.54 (1.20–10.27)	1.10 ± 0.60 0.54 (0.18–2.34)

OMNI-RPE = OMNI rating of perceived exertion; sOMNI-TL = session OMNI training load; HR = heart rate; PV = peak velocity; CV = coefficient of variation. One athlete withdrew, and GNSS data missing for one athlete ^1^; GNSS data missing for one athlete ^2^; HR data missing for one athlete and GNSS data missing for one athlete ^3^.

**Table 2:** Correlations between sOMNI-TL and Edward’s TRIMP, time spent in 0–25%, >25–≤50%, >50–≤70% and >70–100% peak velocity for the easy and hard simulated team game sessions and the team game session.

	Edward’s TRIMP	Time Spent in 0–25% PV	Time Spent in >25–≤50% PV	Time Spent in >50–≤70% PV	Time Spent in >70–≤100% PV
Easy simulated team game ^1^ sOMNI-TL	r = 0.20 [−0.29, 0.62] *p* = 0.401	r = −0.25 [−0.59, 0.21] *p* = 0.327	r = 0.42 [−0.131, 0.731] *p* = 0.083	ρ = 0.30 [−0.26, 0.68] *p* = 0.22	r = 0.05 [−0.34, 0.41] *p* = 0.854
Hard simulated team game ^2^ sOMNI-TL	r = 0.18 [−0.27, 0.56] *p* = 0.453	r = −0.17 [−0.56, 0.31] *p* = 0.474	r = 0.20 [−0.31, 0.63] *p* = 0.415	r = 0.41 [−0.073, 0.682] *p* = 0.078	r = −0.23 [−0.62, 0.16] *p* = 0.348
Team Game ^3^ sOMNI-TL	r = 0.53 * [0.25, 0.76] *p* = 0.001	r = 0.42 * [0.080, 0.72] *p* = 0.015	ρ = 0.50 * [0.180, 0.725] *p* = 0.003	r = 0.51 * [0.297, 0.712] *p* = 0.002	r = 0.36 * [0.07, 0.61] *p* = 0.042

* Correlation is significant at the 0.05 level (2-tailed); r = Pearson’s Product–Moment Correlation Coefficient; [95% confidence interval] ρ = Spearman rank correlation; [95% confidence interval] CI = confidence interval; sOMNI-TL = session OMNI training load; PV = peak velocity. One athlete withdrew and GNSS data missing for one athlete ^1^; GNSS data missing for one athlete ^2^; HR data missing for one athlete and GNSS data missing for one athlete ^3^.

## Data Availability

Data from this study is stored in a secure Curtin University repository and is available upon request.

## Abbreviations

The following abbreviations are used in this manuscript:

TL	Training load
HR	Heart rate
TRIMP	Training impulse
RPE	Rating of perceived exertion
sRPE	Session Rating of perceived exertion
CR	Category ratio
GNSS	Global navigation satellite system
cm	Centimetres
kg	Kilograms
au	Arbitrary units
min	Minutes
PV	Peak velocity
CI	Confidence interval
SD	Standard deviation
CV	Coefficient of variation
NCAA	National Collegiate Athletic Association

## Appendix A. Standardised Warm up and Cool Down

**Table A1:** Standardised warm up (10 min).

Light jog	3 min
Dynamic agility (skipping high knees, skipping heel flicks, walking hamstring sweep, walking groin stretches)	3 min
Fast running/sprints 20 m 1 × 70% of maximum perceived speed 1 × 80% of maximum perceived speed 1 × 90% of maximum perceived speed	2 min
Dynamic stretches Leg swings (forward/backward) 2 × 6 reps Leg swings (side to side) 2 Standardised cool down 6 reps	2 min

**Table A2:** Standardised cool down (10 min).

Walking	5 min
Static Stretches Calves, Hamstrings, Quadriceps, Hip Flexors, Groin, lower back	5 min
